# Factors Affecting the Utilization of a Minimum of Four Antenatal Care Services in Ethiopia

**DOI:** 10.1155/2019/5036783

**Published:** 2019-08-14

**Authors:** Garoma Wakjira Basha

**Affiliations:** Department of Statistics, College of Science, Bahir Dar University, Bahir Dar, Ethiopia

## Abstract

**Background:**

Antenatal care is defined as the routine care of pregnant women provided between conception and the onset of labor. This study is aimed to identify factors affecting the utilization of antenatal care (ANC) services in Ethiopia.

**Methods:**

The study used data from the nationally representative 2016 Ethiopia Demographic and Health Survey (EDHS). A total of 7,167 mothers who gave birth within five years preceding the 2016 EDHS whose complete information was available in the survey were included in this study. Logistic regression statistical analyses were used to identify factors associated with the utilization of a minimum of 4 ANC services in Ethiopia.

**Results:**

Among the 7,167 women included in this study, 2,598 (36.6%) had utilized a minimum of 4 ANC services in Ethiopia. This study showed that factors such as place of residence, region, mothers' education level, household wealth index, desire for pregnancy, frequency of reading newspaper, frequency of listening to radio, and frequency of watching TV were associated with the utilization of a minimum of four ANC services at 5% level of significance in Ethiopia.

**Conclusion:**

Strategies to increase the accessibility and availability of healthcare services are important particularly for communities in rural areas. Financial support that enables mothers from poor households to use health services will be beneficial. Health promotion programs targeting mothers with no education are vital to increase their awareness about the importance of antenatal services.

## 1. Background

The health of women and children remains an unfinished agenda and a global challenge. Efforts and investments are needed to sustain and accelerate progress if countries and the international community are to prevent maternal and child morbidity and reach the related Sustainable Development Goals (SDGs) [1–3].

Antenatal care is defined as the routine care of pregnant women provided between conception and the onset of labor. Antenatal care is an opportunity to provide care for prevention and management of existing and potential causes of maternal and newborn mortality and morbidity [4].

Maternal mortality is unacceptably high. About 830 women die from pregnancy- or childbirth-related complications around the world every day. It was estimated that, in 2015, roughly 303,000 women died during and following pregnancy and childbirth. The high number of maternal deaths in some areas of the world reflects inequities in access to health services and highlights the gap between rich and poor. Almost all maternal deaths (99%) occur in developing countries. More than half of these deaths occur in sub-Saharan Africa and almost one-third occur in South Asia [5–7].

Antenatal care is the care provided by healthcare professionals during pregnancy. Globally, while 85 percent of pregnant women access antenatal care with skilled health personnel at least once, only six in ten (58 per cent) receive at least four antenatal visits. In regions with the highest rates of maternal mortality, such as sub-Saharan Africa and South Asia, even fewer women received at least four antenatal visits (49 per cent and 42 per cent, respectively). Regular contact with a doctor, nurse, or midwife during pregnancy allows women to receive services vital to their health and that of their future children. The World Health Organization (WHO) recommends a minimum of four antenatal care visits. However, global estimates indicate that only about half of all pregnant women receive this recommended amount of care [8].

Antenatal care can help women prepare for delivery and understand warning signs during pregnancy and childbirth. It can be a source of micronutrient supplementation, treatment of hypertension to prevent eclampsia, immunization against tetanus, and HIV testing, in addition to medications to prevent mother-to-child transmission of HIV in cases of HIV-positive pregnant women. In areas where malaria is endemic, health personnel can also provide pregnant women with medications and insecticide-treated mosquito nets to help prevent this debilitating and sometimes deadly disease [8].

The number of women dying due to complications during pregnancy and childbirth has decreased by 43% from an estimated 532,000 in 1990 to 303,000 in 2015. The progress is notable, but the annual rate of decline is less than half of what is needed to achieve the Millennium Development Goal (MDG) target of reducing the maternal mortality ratio by 75% between 1990 and 2015, which would require an annual decline of 5.5%. The 44% decline since 1990 translates into an average annual decline of just 2.3%. Between 1990 and 2000, the global maternal mortality ratio decreased by 1.2% per year, while from 2000 to 2015 progress accelerated to a 3.0% decline per year [9].

Estimates from EDHSs indicate a substantial decline in the pregnancy-related mortality ratio in Ethiopia since 2000, from 871 deaths per 100,000 live births in the 7 years before the 2000 EDHS to 673 deaths per 100,000 live births in the 7 years before the 2005 EDHS, 676 deaths per 100,000 live births in the 7 years before the 2011 EDHS, and 412 deaths per 100,000 live births in the 7 years before the 2016 EDHS [10].

In Ethiopia, even if there is improvement in maternal healthcare service utilization including antenatal care, most of the women did not attend a minimum number of visits recommended by the World Health Organization (WHO). While adequate care during pregnancy and delivery is essential, healthcare service utilization is extremely low. Most of the previous studies conducted in Ethiopia reflected a low utilization of antenatal care in the towns and city [11, 12].

According to the 2016 Ethiopia Demographic and Health Survey report, 62 percent of women who gave birth in the five years preceding the survey received antenatal care from a skilled healthcare provider at least once for their last birth and only 32 percent had four or more ANC visits for their most recent live birth. Among regions, ANC coverage from a skilled provider is highest in Addis Ababa (97%) followed by Tigray (90%) and lowest in Somali (44%) [10]. Hence, this study is aimed to assess factors associated with the utilization of a minimum of four antenatal care services in Ethiopia.

## 2. Methods

### 2.1. Source of Data

This study used data from the 2016 Ethiopia Demographic and Health Survey (EDHS) which is openly available from the Measure DHS website (https://dhsprogram.com).

### 2.2. Study Variables

#### 2.2.1. Response (Outcome) Variable

The outcome variable of this study is “utilization of a minimum of 4 ANC services” during pregnancy, which can be recorded as binary (1 = yes and 0 = no).

#### 2.2.2. Explanatory Variables

The explanatory variables are as follows: place of residence, mothers' education, region, wealth index, age of mothers, mothers' current working status, birth order, desire for pregnancy, frequency of reading newspaper, frequency of listening to radio, and frequency of watching TV.

## 3. Statistical Analysis

The data were analyzed using Statistical Package for Social Science (SPSS) version 21. Frequency and percentage were calculated to describe the characteristics of respondents included in this study. Logistic regression model was used to identify factors associated with the utilization of a minimum of 4 ANC services during pregnancy in Ethiopia. Bivariate logistic regression was carried out between the selected factors and the outcome variable (minimum of 4 ANC visits during pregnancy). Those factors which were significant (i.e., with a *p* value <0.25) in the bivariate logistic regression were selected and retained in the multiple logistic regression model. Multiple logistic regression analysis was conducted to assess the net effect of these factors on a minimum of 4 ANC visits during pregnancy.

## 4. Results

A total of 7,167 mothers who gave birth within five years preceding the 2016 EDHS whose complete information was available in the survey were included in this study. Out of this, 2,598 (36.2%) mothers had utilized a minimum of 4 ANC services during pregnancy.  Table 1 shows that the majority of women residing in rural area (89.2%), women with no education (71.6%), women of poor household (61.7%), and women who were not working currently (73.6%) had not utilized a minimum of 4 ANC visits during pregnancy. Among regions, a minimum of 4 ANC visit was highest in Tigray region (16.6%) and the lowest in Somali region (3.9%). More than half (52.6%) of the women in the age group 25–34 had utilized a minimum of 4 ANC services during pregnancy. About 20.6% of women who gave birth to first-order child had utilized a minimum of 4 ANC services. 79.6% of women who were interested in the birth had then utilized a minimum of 4 ANC services during pregnancy compared to only 5.1% of women who were no more interested in the birth. Women who were not getting information from newspaper (96.5 %), radio (82.0%), and TV (86.5%) at all had not utilized a minimum of 4 ANC services during pregnancy.

### 4.1. Factors Associated with the Utilization of a Minimum of 4 ANC Services

Bivariate logistic regression was used to examine the individual effects of each of the selected factors on the utilization of a minimum of 4 ANC services during pregnancy in Ethiopia. The results of bivariate logistic regression showed that all the factors except birth order were significantly associated with the utilization of a minimum of 4 ANC services at 5% level of significance (Table 2). Then, these factors were included in multiple logistic regression analysis to assess their net effect on the utilization of a minimum of 4 ANC services in Ethiopia. Table 2 shows that compared to women residing in urban area, women residing in rural area were 0.63 times less likely to utilize a minimum of 4 ANC services (adjusted odds ratio (aOR) = 0.63, 95% confidence interval (CI): 0.52–0.77, *p* < 0.001).

Compared to women living in Tigray region, women living in the rest regions of the country except Addis Ababa and Dire Dawa were less likely in utilizing a minimum of 4 ANC services. Controlling for other variables in the model, women with primary education were 1.53 times more likely to utilize a minimum of 4 ANC services (aOR = 1.53; 95% CI: 1.33–1.76; *p* < 0.001) compared to uneducated women, and women with secondary and higher education were 2.14 times more likely to utilize a minimum of 4 ANC services (aOR = 2.14; 95% CI: 1.72–2.67; *p* < 0.001) compared to uneducated women.

Women with middle and rich household wealth index had higher odds of utilizing a minimum of 4 ANC services compared to women with poor household wealth index (aOR = 1.55; 95% CI: 1.31–1.83; *p* < 0.001 and aOR = 2.10; 95% CI: 1.80–2.46; *p* < 0.001, respectively).

Women who were no more interested in the birth were less likely to utilize a minimum of 4 ANC services during pregnancy compared to women who were interested in the birth then (aOR = 0.67; 95% CI: 0.53–0.86; *p* < 0.001).

Compared to women who did not read a newspaper at all, women who read a newspaper less than once a week were 1.31 times more likely to utilize a minimum of 4 ANC services (aOR = 1.31; 95% CI: 1.00–1.72; *p* < 0.05). Women who listen to radio less than once a week were more likely to utilize a minimum of 4 ANC services (aOR = 1.34; 95% CI: 1.12–1.60; *p* < 0.001) and women who listen to radio at least once a week were more likely to utilize a minimum of 4 ANC services (aOR = 1.47; 95% CI: 1.22–1.76; *p* < 0.001) compared to women who did not listen to radio at all. Women who watch TV at least once a week were 1.36 times more likely to utilize a minimum of 4 ANC services (aOR = 1.36; 95% CI: 1.09–1.71; *p* < 0.01) compared to women who did not watch TV at all.

## 5. Discussion

The objective of this study was to assess factors associated with the utilization of a minimum of 4 ANC services in Ethiopia using the nationally representative 2016 EDHS data. A total of 7,167 mothers who give birth within five years preceding the 2016 EDHS whose complete information was available in the survey were included in this study. Out of this, 2,598 (36.2%) mothers had utilized a minimum of 4 ANC services in Ethiopia.

The result of this study showed that women from the rural area were less likely to utilize a minimum of 4 ANC services compared to women from the urban area in Ethiopia. This might be due to inequalities in the health facilities and the awareness of women on ANC services in urban and rural areas. This result was consistent with other studies [13–15] conducted elsewhere.

The finding of this study showed that compared to women from Tigray region, women from the rest regions of Ethiopia except Addis Ababa and Dire Dawa were less likely in utilizing a minimum of 4 ANC services. This might be due to the difference in the health facility among regions of the country. The result was in line with other studies [15, 16].

Consistent with the study conducted in the Hadiya zone, southern Ethiopia [11] and Swaziland [17], the result of this study showed that women with primary, secondary, and higher education were more likely to utilize a minimum of 4 ANC services compared to women with no education in Ethiopia. This may be due to the fact that the more educated the women, the more aware she is on the importance of antenatal care services on her health and newborn child. Also, education empowers women to make a decision to seek health care and enable to identify danger signs of pregnancy complication.

The result of the current study revealed that women with medium and rich household wealth index were more likely to utilize a minimum of 4 ANC services compared to women with poor household wealth index. Women with medium and rich household wealth index were more likely to be able to pay for care-seeking costs such as transportation, medications, and any associated costs. The result was consistent with studies [15, 18, 19].

Desire for pregnancy was found to be significantly associated with the utilization of a minimum of 4 ANC services in Ethiopia. The result of this study showed that women with an unwanted pregnancy were less likely to utilize a minimum of 4 antenatal care services. This may be due to the fact that women with unwanted pregnancy give low value to the child and did not seek skilled birth assistance during pregnancy. The result was in line with the other study [20].

Having access to information through modern media could influence women's knowledge about delivery risks and availability of services [7, 15]. In this study, women who read newspaper, listen to radio, and watch TV at least once a week were more likely to utilize a minimum of 4 ANC services compared to women who did not read newspaper, listen to radio, and watch TV at all. This might be due to the fact that the mass media may increase the knowledge and practice of the women on the role of maternal health care for the health of the mother and newborn babies.

### 5.1. Strengths and Limitation

The current study used a nationally representative 2016 Ethiopia Demographic and Health Survey data. Therefore, it indicates national-level factors of utilization of a minimum of 4 ANC services in Ethiopia. Since the current study used the recent EDHS data, it indicates the updated information on factors of utilization of a minimum of 4 ANC services in Ethiopia. Even though previous studies have identified risk factors of utilization of ANC services, there are limited or no studies on risk factors of a minimum of 4 ANC services utilization recommended by the WHO, specifically in Ethiopia. This study may be used for further study. The limitation of the current study is that the information used is subject to recall bias, as information collected relied on the women's recall ability about her pregnancy.

## 6. Conclusion

The results of this study revealed that factors such as place of residence, region of residence, mother's level of education, household wealth index, desire for pregnancy, and exposure to mass media, newspaper, radio, and TV were significantly associated with the utilization of a minimum of 4 ANC services in Ethiopia. Strategies to increase the accessibility and availability of healthcare services are important particularly for communities in rural areas. A community-based health insurance in Ethiopia needs to implement a policy that adequately mobilizes domestic resources to address the rural women with financial constraints, improves access to healthcare services, and increases the quality of services provided. Since women with poor household wealth index were less likely in utilizing a minimum of 4 ANC services in this study, financial support that enables mothers from poor households to use health services will be beneficial. Health promotion programs targeting mothers with no education are vital to increase their awareness about the importance of antenatal services. Uplifting the socioeconomic status and literacy rate of women is required to provide community-based education.

## Figures and Tables

**Table 1:** Baseline characteristics of minimum of 4 ANC visits during pregnancy in Ethiopia, 2016 EDHS.

Variables	Minimum of 4 ANC visits		
	Yes, *N* (%)	No, *N* (%)	Total, *N* (%)
*Residence*			
Urban	1011 (38.9)	495 (10.8)	1506 (21.0)
Rural	1587 (61.1)	4074 (89.2)	5661 (79.0)

*Region*			
Tigray	430 (16.6)	333 (7.3)	763 (10.6)
Afar	106 (4.1)	539 (11.8)	645 (9.0)
Amhara	231 (8.9)	532 (11.6)	763 (10.6)
Oromia	232 (8.9)	798 (17.5)	1030 (14.4)
Somali	101 (3.9)	698 (15.3)	799 (11.1)
Benishangul	231 (8.9)	345 (7.6)	576 (8.0)
SNNPR^a^	350 (13.5)	540 (11.8)	890 (12.4)
Gambella	185 (7.1)	348 (7.6)	533 (7.4)
Harari	147 (5.7)	263 (5.8)	410 (5.7)
Addis Ababa	334 (12.9)	40 (0.9)	374 (5.2)
Dire Dawa	251 (9.7)	133 (2.9)	384 (5.4)

*Mothers' education level*			
None	1072 (41.3)	3270 (71.6)	4342 (60.6)
Primary	896 (34.5)	1040 (22.8)	1936 (27.0)
Secondary and higher	630 (24.2)	259 (5.7)	889 (12.4)

*Age of mothers*			
15–24	669 (25.8)	1178 (25.8)	1847 (25.8)
25–34	1366 (52.6)	2164 (47.4)	3530 (49.3)
35–49	563 (21.7)	1227 (26.9)	1790 (25.0)

*Wealth index*			
Poor	777 (29.9)	2819 (61.7)	3596 (50.2)
Middle	346 (13.3)	677 (14.8)	1023 (14.3)
Rich	1475 (56.8)	1073 (23.5)	2548 (35.6)

*Respondents currently working*			
No	1654 (63.7)	3362 (73.6)	5016 (70.0)
Yes	944 (36.3)	1207 (26.4)	2151 (30.0)

*Birth order*			
First	535 (20.6)	908 (19.9)	1443 (20.1)
2-3	817 (31.4)	1460 (32.0)	2277 (31.8)
4-5	583 (22.4)	1065 (23.3)	1648 (23.0)
6 or more	663 (25.5)	1136 (24.9)	1799 (25.1)

*Desire for pregnancy*			
Then	2068 (79.6)	3650 (79.9)	5718 (79.8)
Later	397 (15.3)	592 (13.0)	989 (13.8)
No more	133 (5.1)	327 (7.2)	460 (6.4)

*Frequency of reading newspaper*			
Not at all	2156 (83.0)	4411 (96.5)	6567 (91.6)
Less than once a week	343 (13.2)	117 (2.6)	460 (6.4)
At least once a week	99 (3.8)	41 (0.9)	140 (2.0)

*Frequency of listening to radio*			
Not at all	1581 (60.9)	3745 (82.0)	5326 (74.3)
Less than once a week	486 (18.7)	435 (9.5)	921 (12.9)
At least once a week	531 (20.4)	389 (8.5)	920 (12.8)

*Frequency of watching television*			
Not at all	1540 (59.3)	3950 (86.5)	5490 (76.6)
Less than once a week	289 (11.1)	336 (7.4)	625 (8.7)
At least once a week	769 (29.6)	283 (6.2)	1052 (14.7)

SNNPR, Southern Nations, Nationalities, and People's Region.

**Table 2:** Unadjusted and adjusted odds ratio (OR) for factors associated with the utilization of a minimum of 4 ANC services in Ethiopia, 2016 EDHS.

Variables	Unadjusted OR (95% CI)	Adjusted OR (95% CI)
*Residence*		
Urban (ref)	1.00	1.00
Rural	0.19 (0.17–0.22)^*∗∗∗*^	0.63 (0.52–0.77)^*∗∗∗*^

*Region*		
Tigray (ref)	1.00	1.00
Afar	0.15 (0.12–0.20)^*∗∗∗*^	0.20 (0.15–0.26)^*∗∗∗*^
Amhara	0.34 (0.27–0.42)^*∗∗∗*^	0.36 (0.28–0.45)^*∗∗∗*^
Oromia	0.23 (0.18–0.28)^*∗∗∗*^	0.21 (0.17–0.26)^*∗∗∗*^
Somali	0.11 (0.09–0.14)^*∗∗∗*^	0.13 (0.10–0.17)^*∗∗∗*^
Benishangul	0.52 (0.42–0.65)^*∗∗∗*^	0.60 (0.48–0.76)^*∗∗∗*^
SNNPR	0.50 (0.41–0.61)^*∗∗∗*^	0.50 (0.40–0.61)^*∗∗∗*^
Gambella	0.41 (0.33–.52)^*∗∗∗*^	0.35 (0.27–0.45)^*∗∗∗*^
Harari	0.43 (0.34–0.55)^*∗∗∗*^	0.23 (0.18–.30)^*∗∗∗*^
Addis Ababa	6.47 (4.52–9.25)^*∗∗∗*^	1.32 (0.88–1.96)
Dire Dawa	1.46 (1.13–1.90)^*∗∗*^	1.01 (0.76–1.35)

*Mothers' education level*		
None	1.00	1.00
Primary	2.63 (2.35–2.94)^*∗∗∗*^	1.53 (1.33–1.76)^*∗∗∗*^
Secondary and higher	7.42 (6.32–8.71)^*∗∗∗*^	2.14 (1.72–2.67)^*∗∗∗*^

*Age of mothers*		
15–24 (ref)	1.00	1.00
25–34	1.11 (0.99–1.25)	1.12 (0.97–1.29)
35–49	0.81 (0.70–0.93)^*∗∗*^	0.97 (0.82–1.15)

*Wealth index*		
Poor (ref)	1.00	1.00
Middle	1.85 (1.59–2.16)^*∗∗∗*^	1.55 (1.31–1.83)^*∗∗∗*^
Rich	4.99 (4.46–5.58)^*∗∗∗*^	2.10 (1.80–2.46)^*∗∗∗*^

*Respondent currently working*		
No (ref)	1.00	1.00
Yes	1.59 (1.43–1.76)^*∗∗∗*^	1.01 (0.89–1.14)

*Birth order*		
First	1.00	
2-3	0.95 (0.83–1.09)	
4-5	0.93 (0.80–1.08)	
6 or more	0.99 (0.86–1.14)	

*Desire for pregnancy*		
Then (ref)	1.00	1.00
Later	1.18 (1.03–1.36)^*∗*^	0.84 (0.71–0.99)^*∗*^
No more	0.72 (0.58–0.88)^*∗∗*^	0.67 (0.53–0.86)^*∗∗*^

*Frequency of reading newspaper*		
Not at all (ref)	1.00	1.00
Less than once a week	6.00 (4.83–7.44)^*∗∗∗*^	1.31 (1.00–1.72)^*∗*^
At least once a week	4.94 (3.42–7.14)^*∗∗∗*^	1.15 (0.74–1.79)

*Frequency of listening to radio*		
Not at all (ref)	1.00	1.00
Less than once a week	2.65 (2.30–3.05)^*∗∗∗*^	1.34 (1.12–1.60)^*∗∗∗*^
At least once a week	3.23 (2.80–3.73)^*∗∗∗*^	1.47 (1.22–1.76)^*∗∗∗*^

*Frequency of watching television*		
Not at all (ref)	1.00	1.00
Less than once a week	2.21 (1.87–2.61)^*∗∗∗*^	1.04 (0.85–1.28)
At least once a week	6.97 (6.01–8.09)^*∗∗∗*^	1.36 (1.09–1.71)^*∗∗*^

Significance at ^*∗*^*p* < 0.05, ^*∗∗*^*p* < 0.01, and ^*∗∗∗*^*p* < 0.001. aOR: adjusted odds ratio.

## Data Availability

The data used to support the findings of this study are available from the corresponding author upon request.

## Abbreviations

ANC: Antenatal care
EDHS: Ethiopia Demographic and Health Survey
WHO: World Health Organization
SDGs: Sustainable Development Goals
OR: Odds ratio
aOR: Adjusted odds ratio.
